# Associations of betatrophin levels with irisin in Chinese women with normal glucose tolerance

**DOI:** 10.1186/s13098-015-0019-2

**Published:** 2015-03-25

**Authors:** Xinmiao Xie, Ting Gao, Meili Yang, Peihong Chen, Hua Jin, Lili Yang, Xuemei Yu

**Affiliations:** Department of Endocrinology and Metabolism, Fengxian Central Hospital, Shanghai, 201499 China; The Third Clinical Medical College of Southern Medical University, Guangzhou, 510515 China; Ningyang First Hospital, Ningyang, Shandong Province 271400 China

**Keywords:** Betatrophin, Irisin, Type 2 diabetes mellitus, Normal glucose tolerance

## Abstract

**Background:**

Betatrophin may increase islet β cell proliferation in insulin resistance and irisin may improve glucose tolerance in mice. To examine the relationship between betatrophin and irisin, we investigated it in middle-aged Chinese subjects with normal glucose tolerance (NGT) and type 2 diabetes mellitus (T2DM).

**Methods:**

A total of 460 permanent residents of Fengxian District, aged 40–60 years and without T2DM, were enrolled. Anthropometric parameters, oral glucose tolerance test (OGTT) results, glycosylated haemoglobin levels, blood lipid levels, insulin sensitivity (homeostasis model assessment of insulin resistance, HOMA-IR), β cell function (homeostasis model assessment-β, HOMA-β), estimated glomerular filtration rate (eGFR) and body fat composition were determined. Matched for age, gender and body mass index (BMI, 18–28 kg/m2), newly diagnosed T2DM (n = 50, male/female = 23/27) and NGT (n = 50, male/female = 21/29) subjects were selected based on the results of an OGTT. Serum betatrophin and irisin levels were determined by enzyme linked immune sorbent assay (ELISA).

**Results:**

Males had higher levels of betatrophin compared with females in both the NGT and T2DM groups. Compared with NGT subjects, the level of betatrophin in the T2DM group was higher, and males in the T2DM group had higher betatrophin levels than males in the NGT group, but there was no significant difference in betatrophin levels in females between the T2DM and NGT groups. Spearman’s correlation analysis revealed that serum betatrophin levels in females with NGT were positively correlated with irisin and negatively correlated with FINS (fasting insulin) levels ( *p* < 0.05), but no correlation was found between betatrophin and irisin levels in males with NGT or in males or females with T2DM. In females with T2DM, circulating betatrophin levels were positively correlated with weight, BMI and hip circumference (*p* < 0.05) but negatively correlated with FPG (fasting plasma glucose) and HOMA-IR (*p* < 0.05).

**Conclusions:**

Gender differences in the relationship between betatrophin and irisin indicate that there might be cytokine-mediated crosstalk among the liver, adipose tissue and skeletal muscle.

## Background

Diabetes mellitus is one of the most common chronic diseases, and its incidence gradually increases each year. The world prevalence of diabetes among adults was 6.4%, affecting 285 million adults, in 2010, and it is predicted to increase to 7.7% (i.e., 439 million adults) by 2030 [1]. The prevalence of diabetes was estimated at 11.6% in the Chinese adult population [2], and China may be home to the world’s largest population of people living with diabetes. The main mechanisms associated with type 2 diabetes are pancreatic beta cell dysfunction and insulin resistance. Despite extensive research, the mechanisms are not fully understood, and pharmacological treatments targeting insulin resistance and beta cell function are almost non-existent.

Betatrophin (also known as angiopoietin-like protein 8 (ANGPTL8), hepatocellular carcinoma-associated protein-TD26, refeeding-induced fat and liver protein (RIFL) and lipasin) is a newly identified hormone [3-6]. It has been demonstrated that betatrophin, mainly expressed in liver and adipose tissue in mice, promotes pancreatic beta cell proliferation and improves metabolic control [5,7,8]. Recently, Gusarova et al. showed that Angptl8 (−/−) mice does not affect the compensatory proliferation of pancreatic beta cells in response to insulin resistance resulting from either a high-fat diet or the administration of the insulin receptor antagonist S961 [9]. And Yi et al. repeated the experiment in order to find the likely cause of this deviation; finally they obtained results that some mice respond strongly to ANGPTL8/betatrophin expression but many do not, one possible explanation is that ANGPTL8 requires a binding partner that can be induced by tail vein injection; another approach to investigate this issue is to find and investigate the function of the receptor for Angptl8/betatrophin or its protein complex [10]. In all, whether betatrophin does control pancreatic β cell proliferation or not needs further study.

Betatrophin is also involved in lipid metabolism; its overexpression increases serum triglyceride (TG) levels and inhibits the activity of lipoprotein lipase (LPL) [4]. Additionally, Ren G et al. observed that betatrophin knockout mice have serum TG levels approximately one-third of those in wild-type mice [3] and Hobbs et al. also showed that plasma TG levels were reduced in fed Angptl8^− /−^mice [11]. Serum betatrophin levels were found to be higher in subjects with T2DM in many studies, but there have also been studies that found no difference in betatrophin levels and even decreased levels in obese and T2DM subjects [12-16].

Irisin, a myokine that is produced by proteolytic cleavage of fibronectin type III domain containing protein 5 (FNDC5), is secreted by skeletal muscles and increases with exercise [17]. Exercise activates the expression of peroxisome proliferator-activated receptor-γ coactivator-1α (PGC-1α) and uncoupling protein 1 (UCP1), which stimulate the proton transport chains in the mitochondrial membrane and increase ATP and energy consumption in the form of heat. This process results in weight loss and improved insulin sensitivity [17-19]. Serum irisin levels were found to be reduced in long-term, new-onset and undefined diabetes as well as in metabolic syndrome (MetS) and in individuals with raised-FPG [20-23].

Zhang Y and colleagues found that irisin up-regulated PGC-1α and UCP1, likely mediated by p38 mitogen-activated protein kinase (p38 MAPK) and the extracellular regulated protein kinase (ERK) signalling pathway [24]. Therefore, the p38 MAPK and ERK signalling pathways play an important role in the process of WAT “browning” mediated by irisin. Based on previous research, Sanchis-Gomar et al. proposed a pathway involved in the production of insulin resistance that influences glucose and lipid metabolism and involves betatrophin and irisin [25]. The mechanisms underlying type 2 diabetes include insulin secretion by pancreatic β-cells and insulin resistance, which involves hepatocytes, adipocytes and myocytes. Betatrophin is a cytokine expressed in the liver and adipose tissue that increases islet β cell proliferation in insulin resistance [5,7,8], whereas irisin, which promotes white adipose tissue (WAT) “browning” and improves glucose tolerance in mice, is a myokine as well as an adipokine [17,26]. Thus, there might be cytokine-mediated crosstalk among the liver, adipose tissue and skeletal muscle. Hence, the current study aims to explore the relationship between circulating betatrophin levels and irisin and a potential gender dimorphism with respect to betatrophin levels.

## Methods

### Participants

Between December 2012 and September 2013, a community-based health survey was performed in the Fengxian District of Shanghai, China, to determine the prevalence of DM and prediabetes in adults aged 40 to 60 years. A total of 460 subjects (203 men and 257 women) successively underwent a full evaluation of their glucose tolerance status. Of the 460 individuals, there were 60 newly diagnosed T2DM cases, 254 subjects with NGT and 146 subjects with pre-diabetes. Matched for age, gender and body mass index (BMI, 18–28 kg/m^2^), newly diagnosed T2DM (n = 50, male/female = 23/27) and NGT (n = 50, male/female = 21/29) subjects were selected based on the results of an OGTT [27]. Subjects with a fasting plasma glucose level of ≥ 7.0 mmol/L or a 2-hour plasma glucose level of ≥ 11.1 mmol/L were diagnosed with DM. Subjects with T2DM were not treated with anti-diabetic medications. Individuals with a history of DM, gestational diabetes, acute or chronic inflammatory disorders, cancer, active hepatitis/liver cirrhosis, severe cardiovascular or kidney diseases or other known major diseases were excluded from the study. This study was approved by the Medical Ethics Committee of the Shanghai Fengxian District Central Hospital, and all subjects gave written informed consent.

### Measurements

Height (without shoes) and weight (without shoes and in light clothing) were measured, and BMI (kg/m^2^) was calculated by dividing the subjects’ weight (kg) by their height (m^2^). The subjects stood with their feet 25–30 cm apart for even weight distribution; waist circumference was measured midway between the lower rib margin and the iliac crest at the end of expiration, and the hip circumference measurement was taken around the most prominent point of the pelvis. Blood pressure was measured 3 times using a mercury sphygmomanometer at resting state, and an average value was obtained. Skeletal muscle, body fat mass and body fat percentage were measured with a human body composition analyser (INBODY S10, Korea). All blood samples were taken in the morning following an overnight fast of at least 8 h. After clotting, blood specimens were separated by centrifugation, and serum samples were subsequently stored in aliquots without preservatives at −80°C until analysis of betatrophin and irisin. A 75 g glucose solution was administered orally, and plasma glucose was measured after 2 hours. In all subjects, fasting plasma glucose (FPG), 2-hour plasma glucose (2 h-PG), serum total cholesterol (TC), triglycerides (TG), low-density lipoprotein cholesterol (LDL), high-density lipoprotein cholesterol (HDL), free fatty acids (FFA), blood urea nitrogen (BUN), serum creatinine (Cr), and uric acid (UA) were measured using an automatic biochemical analyser (Beckman DXC800, USA). Haemoglobin A1C (HbA1C) was measured by high-pressure liquid chromatography (TOSOH HLC-723 G7, Japan), and fasting insulin (FINS) was measured in an electrochemiluminescence immunoassay (ADVIA Centaur, Germany). Homeostasis model assessment of insulin resistance (HOMA-IR) values were calculated as FPG (mM) × FINS (mU)/22.5. Homeostasis model assessment-β (HOMA-β) values were calculated as 20 × FINS (mU)/[FPG (mM)- 3.5]. The estimated glomerular filtration rate (eGFR) values were calculated from creatinine levels using the CKD-EPI formula [28]. Serum irisin levels were determined using a commercially available human ELISA kit (PHOENIX PHARMACEUTICALS, INC., USA), and plasma betatrophin levels were determined via ELISA (Wuhan Eiaab Science, Wuhan, China; Catalogue No. E11644h). Intra-assay CV and inter-assay CV of betatrophin were less than 4.8% and 7.2%; while the intra-assay CV and inter-assay CV of irisin were less than 10% and 15%, respectively.

### Statistics

Statistical analysis was performed using SPSS 19.0 software. The data are presented as the mean ± SD or median (interquartile range). Continuous variables that followed normal distributions were compared via t-test, whereas those that did not follow a normal distribution were compared using the Wilcoxon signed-rank test. Correlation coefficients were analysed using Spearman’s correlation. *P* values < 0.05 were considered statistically significant.

## Results

### Characteristics of study participants

The clinical and laboratory characteristics of the study subjects are presented in Table 1. In subjects with NGT, compared with female subjects, the levels of betatrophin, height, weight, BMI, waist circumference, hip circumference, WHR, Cr, UA, TG and FPG were significantly higher among men (p < 0.05). In men with T2DM, betatrophin levels were also significantly higher than in women with T2DM; men also had greater height, weight, BMI, waist circumference, hip circumference, Cr and UA (p < 0.05); however, TC and HDL levels were significantly higher (p < 0.05) in women.

**Table 1:** **Baseline clinical characteristics of subjects with normal glucose tolerance (NGT) and type 2 diabetes mellitus (T2DM)**

**Factor**	**NGT**			**T2DM**		
	**Male**	**Female**	***P***	**Male**	**Female**	***P***
N	21	29		23	27	
Age (years)	53.00 (46.50,57.50)	55.00 (49.50,58.00)	0.903	51.82 ± 6.91	51.65 ± 4.66	0.925
Height (cm)	165.76 ± 8.29	154.81 ± 4.13	<0.001*	165.91 ± 5.21	156.06 ± 5.46	<0.001*
Weight (kg)	69.67 ± 7.36	57.36 ± 5.77	<0.001*	70.65 ± 5.88	58.37 ± 7.71	<0.001*
BMI (kg/m^2^)	25.33 ± 1.56	23.81 ± 2.17	0.009*	25.65 (24.92,26.18)	23.92 (22.76,25.21)	0.003*
Waist (cm)	85.95 ± 4.92	79.91 ± 9.26	0.005*	87.59 ± 4.51	82.79 ± 8.60	0.017*
Hip (cm)	92.14 ± 3.48	89.30 ± 4.40	0.019*	92.80 ± 3.51	89.77 ± 6.51	0.046*
WHR	0.9 3(0.89, 0.98)	0.90 (0.81, 0.94)	0.038*	0.94 ± 0.05	0.92 ± 0.09	0.339
SBP (mmHg)	124.00 (110.00, 134.00)	120.00 (110.00, 130.00)	1.00	130.00 (124.00, 150.00)	140.00 (120.00, 141.00)	0.755
DBP (mmHg)	80.00 (70.00, 90.00)	80.00 (73.00, 85.50)	0.688	90.00 (77.50, 100.00)	83.00 (74.50, 90.00)	0.559
Body fat (kg)	17.48 ± 3.81	19 ± 4.98	0.256	22.49 ± 4.16	20.21 ± 4.52	0.088
BFR (%)	29.05 ± 6.38	30.93 ± 6.71	0.331	32.68 ± 4.56	31.90 ± 5.11	0.596
Muscle mass (kg)	23.74 ± 4.83	23.34 ± 5.25	0.788	25.78 ± 5.49	23.61 ± 3.84	0.140
BUN (mM)	4.76 ± 0.90	5.08 ± 1.77	0.450	4.86 ± 1.00	4.72 ± 1.04	0.627
Cr (uM)	70.10 ± 9.56	54.97 ± 8.75	<0.001*	65.00 ± 11.65	50.70 ± 12.06	<0.001*
UA (uM)	336.24 ± 81.71	223.62 ± 66.61	<0.001*	329.30 ± 78.76	269.67 ± 67.13	0.006*
eGFR (rml/min/1.73 m^2^)	100.86 ± 11.27	102.74 ± 9.04	0.516	107.63 ± 10.29	109.81 ± 14.63	0.551
TC (mM)	5.10 ± 0.85	5.32 ± 1.27	0.484	4.90 (1.18, 3.31)	5.67 (5.45, 5.90)	0.041*
TG (mM)	1.77 ± 1.04	1.08 ± 0.52	0.009*	1.75 (1.16, 3.33)	1.22 (0.96, 1.89)	0.298
HDL (mM)	1.23 ± 0.24	1.37 ± 0.25	0.054	1.24 ± 0.22	1.48 ± 0.54	0.040*
LDL (mM)	2.94 ± 0.60	3.15 ± 0.95	0.387	2.93 ± 0.82	3.24 ± 0.97	0.233
FFA (mM)	0.38 ± 0.19	0.40 ± 0.16	0.643	0.54 ± 0.27	0.82 ± 1.04	0.234
FPG (mM)	5.40 ± 0.42	5.13 ± 0.42	0.027*	7.47(6.40,8.80)	6.80(6.15,9.43)	0.477
2 h-PG (mM)	5.85 ± 1.32	6.33 ± 3.26	0.118	14.65(12.15,17.73)	14.15(12.15,19.73)	0.586
HbA1C (%)	5.55 (5.30, 5.70)	5.40 (5.20, 5.60)	0.206	6.60 (5.80, 8.20)	6.50 (5.90, 8.38)	0.741
FINS (mU/l)	4.15 (3.21, 6.71)	5.67 (3.82, 6.72)	0.254	8.85 (6.26, 11.53)	9.07 (6.47, 12.13)	0.969
HOMA-IR	1.38 ± 0.75	1.71 ± 0.91	0.182	2.82 (2.04, 3.31)	2.77 (2.27, 5.00)	0.633
HOMA-β (%)	27.67 (19.67, 60.77)	35.70 (25.00, 54.10)	0.331	43.25 (30.03, 62.47)	53.64 (33.12, 81.99)	0.430
Betatrophin (pg/ml)	299.61(146.56,439.65)	242.07(67.68,319.41)	0.033*	498.86 ± 239.79	334.59 ± 216.42	0.014*
Irisin (ng/ml)	244.38 (214.18, 305.92)	243.57 (193.49, 347.25)	0.687	249.53 (202.21, 455.08)	241.81 (205.82, 278.94)	0.808

Data are presented as the mean ± SD or median (interquartile range). *,*P*<0.05.

BMI: body mass index; WHR: waist-to-hip ratio; SDP: systolic blood pressure; DBP: diastolic blood pressure; BFR: body fat rate; BUN: blood urea nitrogen; Cr: creatinine; UA: uric acid; eGFR: estimated glomerular filtration rate; TC: total cholesterol; TG: triglyceride; HDL: high-density lipoprotein cholesterol; LDL: low-density lipoprotein cholesterol; FFA: free fatty acids; FPG: fasting plasma glucose; 2 h-PG: 2 h plasma glucose; HbA1C: hemoglobin A1C; FINS: fasting insulin HOMA-IR: homeostasis model assessment of insulin resistance; HOMA-β: homeostasis model assessment-β.

### Gender dimorphism regarding circulating betatrophin and irisin concentrations in subjects with NGT and T2DM

**Figure 1:**
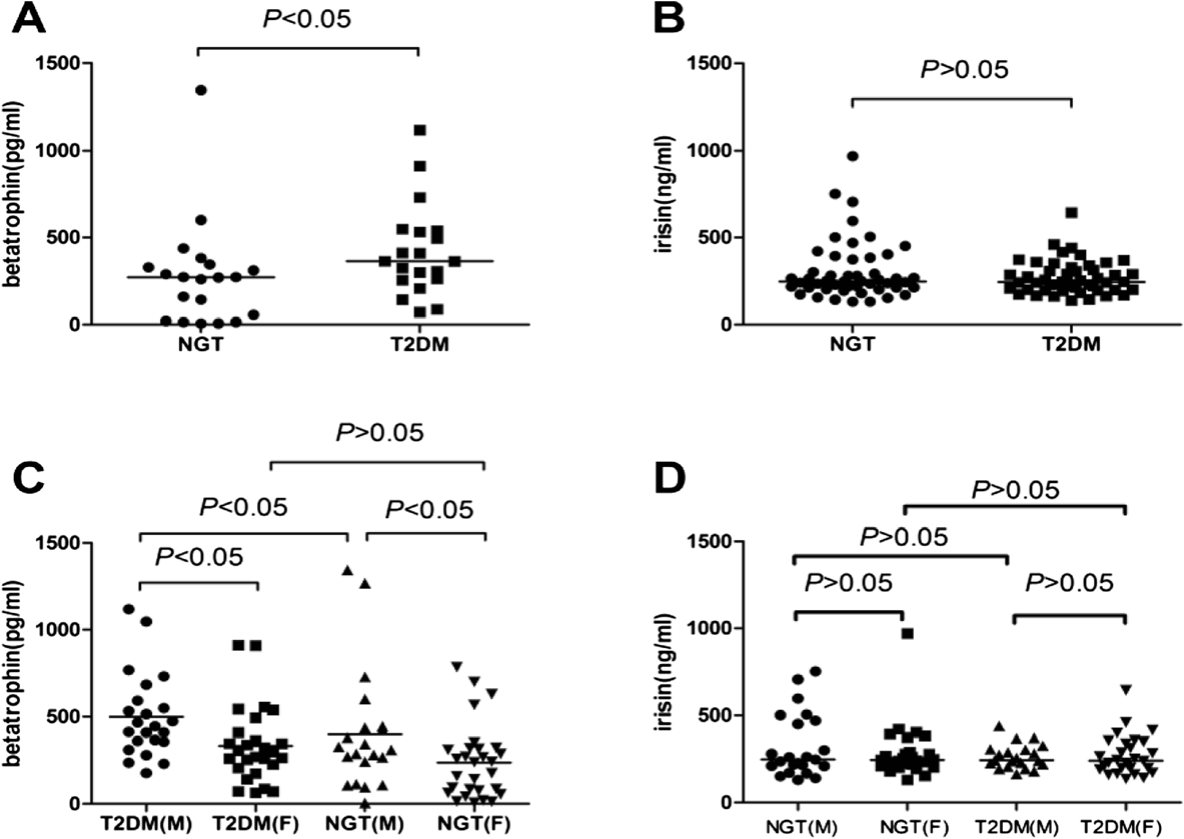
**Gender dimorphism of circulating betatrophin and irisin concentrations in NGT and T2DM. A**. Comparison of serum betatrophin concentrations between NGT and T2DM. **B**. Comparison of serum irisin concentrations between NGT and T2DM. **C**. Comparison of serum betatrophin concentrations between males and females with NGT and T2DM. **D**. Comparison of serum irisin concentrations between males and females with NGT and T2DM.

Compared with subjects with NGT, the level of betatrophin in subjects with T2DM was higher (Figure 1A), and males with T2DM had higher betatrophin levels than males with NGT, but there was no significant difference between females with T2DM and females with NGT (Figure 1C). Men had higher levels of betatrophin compared with women in both the NGT and T2DM groups (Figure 1C). There were no significant differences in irisin levels between the NGT and T2DM groups (Figure 1B). Moreover, no gender difference in circulating irisin levels was found in our study (Figure 1D).

### Correlation of betatrophin with clinical parameters

**Figure 2:**
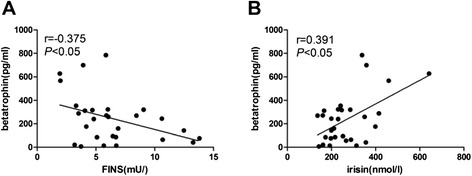
**Correlation analysis to evaluate the correlation of circulating betatrophin with irisin and FINS in women with NGT.** Spearman bivariate correlation analysis showed that FINS (r = -0.375, p <0.05) was negatively correlated with circulating betatrophin and irisin (r=0.391,p<0.05) was positively correlated with circulating betatrophin , the figures were presented in **A** and **B**.

**Figure 3:**
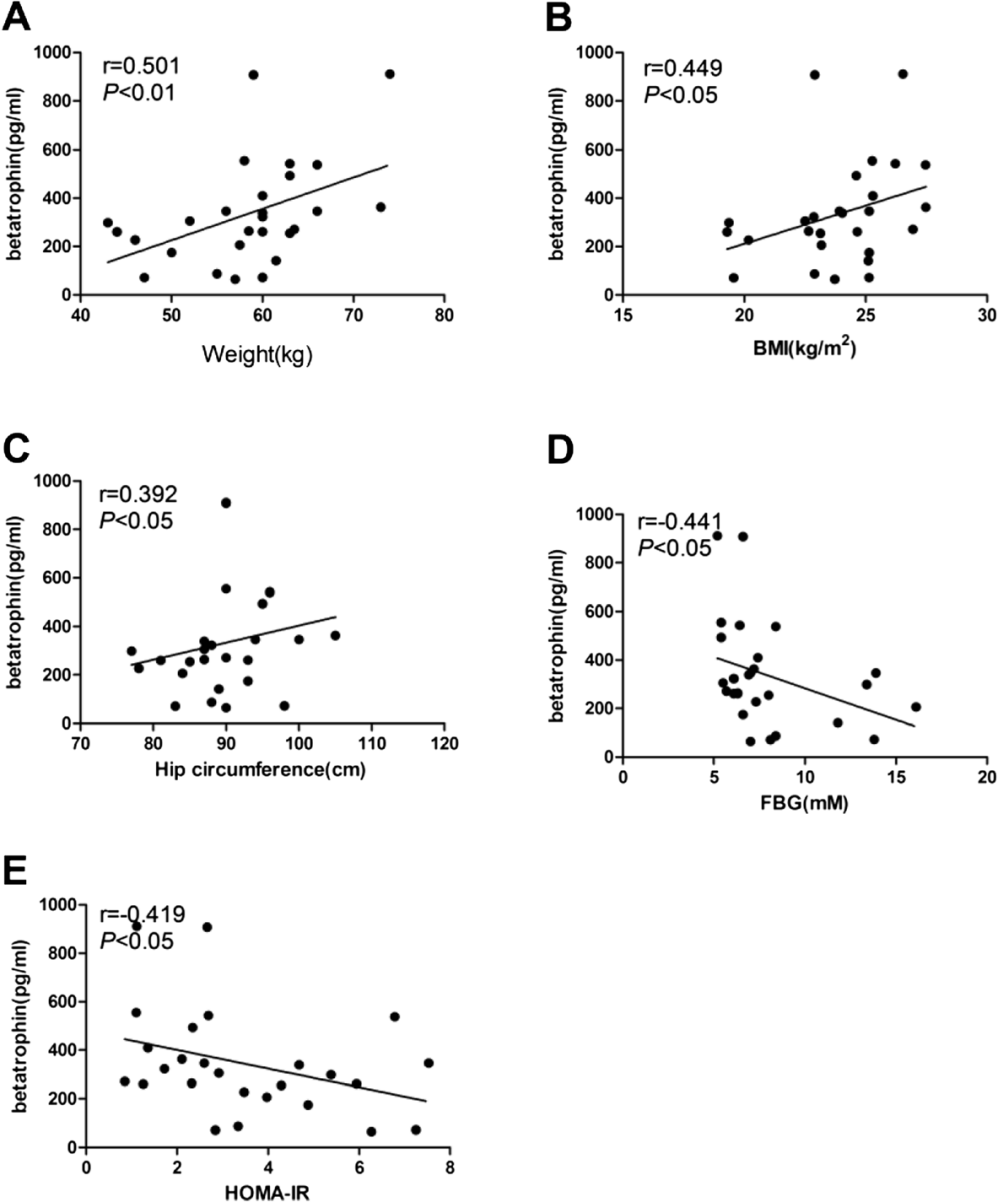
**Correlation analysis to evaluate the correlation of circulating betatrophin with weight, BMI, hip circumference, FBG and HOMA-IR in women with T2DM.** Spearman bivariate correlation analysis showed that weight (r =0.501, p <0.01), BMI (r =0.449, p <0.05) and hip circumference (r=0.392, p<0.05) were positively correlated with circulating betatrophin, the figures were presented in **A**, **B** and **C**; FBG (r=-0.441,p<0.05) and HOMA-IR (r=-0.419,p<0.05) were negatively correlated with circulating betatrophin, the figures were presented in **D** and **E**.

Correlation coefficients between betatrophin levels and the measured clinical parameters are presented in Figures 2 and 3. Spearman’s correlation analysis revealed that serum betatrophin levels in females with NGT were positively correlated with irisin and negatively correlated with FINS (*p* < 0.05) (Figure 2), but no correlation was found between betatrophin and irisin in males with NGT or in males or females with T2DM (data not shown). In females with T2DM, circulating betatrophin levels were positively correlated with weight, BMI and hip circumference but negatively correlated with FPG and HOMA-IR (*p* < 0.05) (Figure 3).

## Discussion

Diabetes is a public health problem throughout the world as well as in China, and it is particularly important to explore treatments that focus on the mechanisms of diabetes—impaired pancreatic beta cell function and insulin resistance. Betatrophin and irisin are two novel hormones that have been intensively investigated in recent diabetes treatment research that potentially take part in the mechanisms of T2DM associated with insulin resistance and β cell function.

Betatrophin has been identified as a novel hormone that belongs to the family of angiopoietin-like proteins and is coded by Gm8464 (in mice) and C19orf80 (in human) [3]. It has been demonstrated that betatrophin is a liver- and fat-derived hormone that promotes pancreatic β cell proliferation and improves glucose tolerance [5]. Espes and colleagues measured betatrophin concentrations in humans for the first time and observed that serum betatrophin levels were approximately doubled in patients with type 1 diabetes compared with controls [29]. Our study demonstrated that circulating betatrophin levels were significantly higher in newly diagnosed T2DM subjects than in NGT subjects; this finding was consistent with the results of Espes’s recent research on T2DM [14] as well as research by Fu et al. [12] and Hu et al. [13]. However, one recent study reported that betatrophin concentrations did not differ between T2DM and non-diabetic participants [15], and another reported decreased betatrophin levels in obese and T2DM subjects [16]. The paradoxical results may be related to the following observations. First, the BMI of participants in the previous study was higher than 30 kg/m^2^, and the participants in the present study had BMIs between 18 and 28 kg/m^2^. Second, all patients with T2DM in Fenzl et al.’s study received medication, and the patients with T2DM in the present study were all newly diagnosed and were not receiving medication. Third, a recent publication suggests that the differences in recent findings on betatrophin should be discussed in light of differences between two commercially available ELISA kits [30], and this may help explain the paradoxical results. Finally, we cannot exclude that increased betatrophin levels may be transient due to functional compensation of pancreatic β cells in early diabetes.

Our data also revealed a sexual dimorphism with respect to betatrophin levels and demonstrated that serum betatrophin concentrations were significantly lower in women than in men. Gómez-Ambrosi et al. [16] were the first to report this dramatic gender dimorphism in betatrophin levels; however, the results they obtained were inconsistent with those of the present study. In their study, higher betatrophin levels were found in females than in males. The differing results may be attributed to differences in test kits or subject characteristics. Fu et al. compared the betatrophin kits manufactured by Eiaab (Catalogue no. E1164H; Wuhan, China) and Phoenix Pharmaceuticals (Catalogue no. EK-051-55; Burlingame, CA, USA) [30]. They found that two kits used different antibodies, which recognized the N- and C-terminus of betatrophin, respectively. They indicated that serum betatrophin levels were positively or negatively correlated with BMI in studies using Eiaab or Phoenix Pharmaceuticals’ kits. Therefore, the use of different kits may help explain the paradoxical results with respect to gender differences in the two studies.

Yi and colleagues found that overexpression of betatrophin in mice causes a 17-fold increase in β cell proliferation, and the specific deletion of β cells with diphtheria toxin did not up-regulate betatrophin, suggesting that betatrophin levels are regulated by insulin resistance but not insulin deficiency [5]. The result that the HOMA-IR in T2DM subjects was higher than that in NGT (data not shown) suggests that insulin resistance in T2DM subjects is more common than in NGT subjects. At the same time, betatrophin levels were related to HOMA-IR in women with T2DM, and these results were consistent with the results of previous animal studies, in which it was observed that betatrophin could improve insulin resistance.

The characteristics of insulin resistance include senility, obesity, higher SBP, low HDL-cholesterol levels, increased TG concentrations and low alcohol intake [31]. Betatrophin levels in T2DM were associated with BMI and waist circumstance, but there was no correlation between betatrophin concentrations and age, SBP, HDL-cholesterol or TG (data not shown). In females with T2DM, circulating betatrophin levels were positively correlated with weight, BMI and hip circumference. These findings were partly inconsistent with the results of a previous study on T2DM. Fenzl et al. observed that betatrophin levels were associated with LDL-cholesterol and TC but not BMI [15]. However, Espes et al. found that age, BMI, SBP, HDL-C and TG were all not associated with betatrophin [14]. The differing results may be attributable to race and BMI differences.

In many studies, serum irisin levels have been found to be reduced in T2DM subjects. Liu et al. studied 96 T2DM and 60 non-diabetic subjects and found that circulating irisin was significantly lower in subjects with long-term T2DM compared with non-diabetic controls [20]. Decreased circulating irisin was also found in subjects with new-onset T2DM by Choi Y.K. [21], in raised-FPG subjects by Yan B. et al. [22] and in undefined T2DM-status subjects by Moreno-Navarrete et al. [23]. However, in our study, there were no significant differences in irisin levels between NGT and T2DM subjects. Moreover, no gender differences in circulating irisin levels were found in our study. Gender differences in irisin levels were also not found in middle-aged adults in a previous study [23]. However, higher irisin levels were previously reported in healthy girls compared with boys [32] and young healthy women compared with men [33]. In another study, obese men had higher irisin than women [34].

Based on previous studies, Sanchis-Gomar et al. hypothesized that there is a pathway in which irisin and betatrophin are involved in insulin resistance [25]. The authors discovered that exercise increases ROS levels and then activates p38 MAPK, which regulates PGC-1α by inducing the expression of FNDC5. FNDC5 cleaves and secretes irisin, which acts on WAT to stimulate UCP1 expression. Furthermore, this activity promotes the expression of betatrophin and β cell proliferation and reduces insulin resistance. Our study demonstrated that betatrophin levels were positively associated with irisin in women with NGT, and this was consistent with the hypothesis proposed by Sanchis-Gomar and her colleague. In addition to supporting this hypothesis, our results suggested the increased likelihood of a p38-PGC-1α–irisin–betatrophin axis in humans. However, our study found no correlation between betatrophin and irisin in women with newly diagnosed T2DM, which might be due to the following reasons: exercise involved in irisin expression and secretion was not evaluated in this study, and diabetes mellitus is a group of metabolic diseases influenced by many factors (such as obesity, blood lipids, insulin, dietary and so on), and we only excluded the confounding factor of obesity in our study.

There were several limitations regarding the design of this study. A major limitation of this study is the small number of patients who were available for analysis. A secondary limitation was its retrospective design. The final limitation was that we were not able, because of funding constraints, to obtain data after OGTT.

## Conclusions

Our study demonstrated that serum betatrophin levels in NGT subjects were lower than in newly diagnosed T2DM subjects and that males had higher levels of betatrophin than females in both the NGT and T2DM groups. Moreover, betatrophin levels were associated with serum irisin and FINS in women with NGT, whereas there was no correlation between betatrophin and irisin in subjects with newly diagnosed T2DM; circulating betatrophin was positively correlated with weight, BMI and hip circumference but negatively correlated with FPG and HOMA-IR. The results indicate that betatrophin and irisin may play a role in the mechanisms underlying T2DM associated with insulin resistance and β cell function, and cytokine-mediated crosstalk may occur among the liver, adipose tissue and skeletal muscle.

## Abbreviations

WAT: White adipose tissue
T2DM: Type 2 diabetes mellitus
NGT: Normal glucose tolerance
OGTT: Oral glucose tolerance test
ANGPTL8: Angiopoietin-like protein 8
RIFL: Refeeding-induced fat and liver protein
TG: Triglyceride
LPL: Lipoprotein lipase
FNDC5: Fibronectin type III domain containing protein 5
PGC-1α: Peroxisome proliferators-activated receptor-γ coactivator-1α
UCP1: Uncoupling protein 1
MetS: Metabolic syndrome
p38 MAPK: p38 mitogen-activated protein kinase
ERK: Extracellular regulated protein kinase
BMI: Body mass index
FPG: Fasting plasma glucose
2h-PG: 2-hour plasma glucose
TC: Total cholesterol
TG: Triglyceride
LDL: Low-density lipoprotein cholesterol
HDL: High-density lipoprotein cholesterol
FFA: Free fatty acids
BUN: Blood urea nitrogen
Cr: Creatinine
UA: Uric acid
eGFR: Estimated glomerular filtration rate
HbA1C: Haemoglobin A1C
FINS: Fasting insulin
HOMA-IR: Homeostasis model assessment of insulin resistance
HOMA-β: Homeostasis model assessment-β
WHR: Waist-hip ratio
SDP: Systolic blood pressure
DBP: Diastolic blood pressure
BFR: Body fat rate
